# Chinese Named Entity Recognition for Dairy Cow Diseases by Fusion of Multi-Semantic Features Using Self-Attention-Based Deep Learning

**DOI:** 10.3390/ani15060822

**Published:** 2025-03-13

**Authors:** Yongjun Lou, Meng Gao, Shuo Zhang, Hongjun Yang, Sicong Wang, Yongqiang He, Jing Yang, Wenxia Yang, Haitao Du, Weizheng Shen

**Affiliations:** 1 College of Electrical Engineering and Information, Northeast Agricultural University, Harbin 150030, China; louyongjun12@163.com (Y.L.); zhang_shuo_2023@163.com (S.Z.); heyongqiang@mengniu.cn (Y.H.); yjing0210@163.com (J.Y.); 15230569226@163.com (W.Y.); 2 Animal Husbandry and Veterinary Institute of Shandong Academy of Agricultural Sciences, Ji’nan 250010, China; longfei1997@sina.com; 3 School of Engineering, Hong Kong University of Science and Technology, Hong Kong 999077, China; 15776623506@163.com; 4 Dairy Association of Heilongjiang Province, Harbin 150030, China; dahlj2007@163.com

**Keywords:** Chinese named entity recognition, dairy cow disease, multi-level features, Bi-LSTM, self-attention

## Abstract

Named entity recognition (NER) is the basic task of constructing a high-quality knowledge graph, which can provide reliable knowledge in the auxiliary diagnosis of dairy cow disease, thus alleviating problems of missed diagnosis and misdiagnosis due to the lack of professional veterinarians in China. Targeting the characteristics of the Chinese dairy cow diseases corpus, we propose an ensemble Chinese NER model incorporating character-level, pinyin-level, glyph-level, and lexical-level features of Chinese characters. These multi-level features were concatenated and fed into the bidirectional long short-term memory (Bi-LSTM) network based on the multi-head self-attention mechanism to learn long-distance dependencies while focusing on important features. Finally, the globally optimal label sequence was obtained by the conditional random field (CRF) model. Experimental results showed that our proposed model outperformed baselines and related works with an F1 score of 92.18%, which is suitable and effective for named entity recognition for the dairy cow disease corpus.

## Simple Summary

Building a high-quality knowledge graph of dairy cow diseases is one of the main concerns in the cattle breeding industry; it can serve as a reliable foundation for subsequent applications, including answering disease-related questions and auxiliary diagnosis systems, which can significantly lower the barrier for farmers and dairy farms to access professional knowledge. The named entity recognition (NER) task is crucial for constructing a knowledge graph and aims to extract key information such as disease names and symptoms from textual data, where the disease name and symptom information are referred to as entities. According to the characteristics of Chinese dairy cow disease texts, this study explored a named entity recognition method based on multi-semantic features. The results show that the proposed model achieved good recognition performance. Our work provides a foundation for the effective utilization of dairy cow disease knowledge in practical applications and a new insight for named entity recognition for other animal or crop diseases.

## 1. Introduction

In the vast rural areas of China, there is a large shortage of professional veterinarians, which often leads to the missed diagnosis and misdiagnosis of dairy cow diseases, resulting in a decline in production performance and even death or elimination due to a lack of timely treatment, and dairy farms have suffered huge economic losses. Currently, there are some methods for dairy cow disease warning through behavior recognition [1,2]. Abnormal behavior may indicate a possible disease; however, disease diagnosis still needs to consider multiple symptoms and conduct comprehensive diagnosis according to professional knowledge. The knowledge graph is one of the most advanced knowledge representation methods; it has been widely used in answering knowledge-based questions [3,4], disease or fault diagnosis [5,6], and fraud detection [7,8] and is able to provide a powerful tool for the auxiliary diagnosis of dairy cow diseases. Named entity recognition (NER) is the core task for constructing a knowledge graph. Accurately identifying related entities, such as disease names, symptoms, pathogens, and medicines, is crucial for building a high-quality knowledge graph for the diagnosis and treatment of dairy cow diseases, which is beneficial for improving the accuracy and efficiency of disease diagnosis, thus reducing the economic losses caused by the shortage of professional veterinarians for dairy farms and improving livestock welfare.

In this paper, we focus on the task of named entity recognition, which has been tackled in many fields such as geography [9], biomedicine [10,11], finance [12,13], agriculture [14,15], etc. However, relatively less attention has been given to livestock and poultry diseases, especially dairy cow diseases. Additionally, there are still some challenges that need to be solved in dealing with the Chinese NER task for dairy cow diseases. (1) Compared to entities in general fields, entities for dairy cow diseases are more complex and diverse. They are usually composed of Chinese characters, letters, and numbers and often contain some professional terms such as “甲氧嘧啶 (methoxypyrimidine)”, “拜耳9015 (Bayer 9015)”, and “磺胺-6-甲氧嘧啶 (Sulfanilamide-6-methoxypyrimidine)”. (2) As they are hieroglyphics, the morphology of Chinese characters is closely related to their meanings. For example, “病 (disease)” and “疫 (epidemic)” not only share the same radical “疒” but also have similar meanings; both refer to disease. “肿 (swell)” and “胀 (inflate)” share the same radical “月 (round)”, and they both refer to symptoms that occur on a certain body part. (3) There may be different expressions for the same entity in different data sources. For example, “瘤胃臌气 (rumen distension)” and “瘤胃鼓气 (rumen distension)” refer to the same disease entity; the difference between them is the characters “臌 (swell)” and “鼓 (bulge)”, while they share the same pronunciation, “gǔ”. (4) There are also many long-distance entities. For example, the symptom entity “粪便稀薄，混有黏液、脓液 (thin stool mixed with mucus and pus)” is a sequence with 12 Chinese characters. These types of entities are extremely common in the Chinese dairy cow disease corpus, which has increased the difficulty of named entity recognition.

Early NER approaches [16,17] were inflexible, in which dictionary-based methods could not promptly cover newly added entities, while rule-based methods rely heavily on hand-crafted rules which are difficult to transfer and generalize. As machine learning-based NER methods [18,19,20] have been proposed, the practicality and portability of NER methods have been greatly enhanced, but they still depend on manually designed features, which limits their generalization capability to a certain extent. Recently, deep learning methods have made significant progress in NER, which are capable of automatically learning features from textual data, thereby simplifying the complex process of manually constructing features.

In Chinese NER tasks, character-based deep learning models are mostly employed due to the potential error propagation caused by word segmentation mistakes. However, they ignore lexical boundary information and the intrinsic semantic information of Chinese characters, which restrict their recognition performance. To address these issues, recent studies have focused on incorporating lexical and glyph information into character-based NER models to enhance the feature representation ability. Regarding the utilization of lexical information, Zhang and Yang [21] proposed the Lattice-LSTM model, which involves a specific lattice structure to encode the words matched by each character in the dictionary, thus improving NER performance. Ma et al. [22] introduced the SoftLexicon model, which integrates lexical information into the character representation layer without altering the encoding layer. Li et al. [23] proposed the FLAT model, which uses a positional encoding method to convert the lattice structure into a flat structure, further promoting the effective use of lexical information. Based on the SoftLexicon model, Zhang et al. [24] introduced a criss-cross attention module (CCAM) and improved SoftLexicon to create AttSoftlexicon to enhance the utilization of lexical information in the kiwi fruit pest and disease domain. In terms of utilizing glyph information, Yin et al. [25] developed the Bi-LSTM-CRF model, incorporating radical features, which performed well in capturing the deep semantic information embedded in Chinese characters. Guo et al. [26] proposed the CG-ANER model, which involves an image feature extraction network based on a 3D CNN to capture textual glyph features and utilizes the pre-trained BERT model to extract contextual semantic features and uses the fused features to recognize agricultural entities. Gu et al. [27] proposed the CGR-NER model, which involves a hybrid neural network combining 3D CNN and Bi-LSTM to extract semantic information and morphological information between adjacent glyphs from Chinese character image sequences.

In addition to utilizing lexical and glyph information, some studies have tried incorporating fine-grained semantic features of Chinese characters, such as pinyin and Wubi, into models to enrich feature representation. Moreover, other researchers have actively explored and integrated multiple semantic features to further enhance recognition performance. Li et al. [28] proposed the RG-FLAT-CRF model, which uses a convolutional neural network (CNN) to capture Chinese character image features, RoBERTa to generate character embeddings, and FLAT to incorporate lexical information, enabling effective named entity recognition in Chinese electronic medical records. Wang et al. [29] incorporated features such as Wubi and character images into the encoding layer to deal with named entity recognition in Chinese electronic medical records. Wu et al. [30] extended the FLAT model by integrating Chinese characters’ structural information, achieving a multi-feature embedding approach that includes both lexical and radical features. In the field of livestock and poultry diseases, Yang and Wang [31] injected lexicon information into character vectors and used a CNN to extract radical features. These features were concatenated and fed into a Bi-LSTM-CRF model for sheep disease NER. Although the above methods have attempted to integrate multi-semantic information into character-based models, most of the research only considers one or two features and has not been able to integrate the glyph information, lexical information, and fine-grained semantic information of Chinese characters into the model together, resulting in the need to improve the textual representation ability of the model to adapt to complex and diverse datasets.

In order to solve the issues in dairy cow disease text, we propose an ensemble model that incorporates character-level, pinyin-level, glyph-level, and lexical-level features of Chinese characters to deal with the named entity recognition task for dairy cow diseases. To tackle the first problem, we employed an external lexicon pre-trained on a domain-specific dairy cow diseases corpus to help recognize the boundaries of entities containing special terms. For the second problem, we utilized a two-dimensional convolutional neural network (2D CNN) to extract rich glyph features from images of Chinese characters in dairy cow disease texts. To address the third problem, we designed a pinyin encoder to convert the text sequence into its corresponding pinyin sequence and used a CNN to extract pinyin features. Finally, to overcome the challenge in dealing with long-distance dependencies, we used a Bi-LSTM with self-attention mechanism to capture representations of important characters for recognizing entities and input them into a CRF to obtain the labels of entity types. Compared to previous studies [24,31], we introduced more features that are in line with the characteristics of Chinese text, making our model stronger in representing dairy cow disease texts. We conducted experiments on a self-constructed dairy cow disease dataset; the results indicate that our proposed model is superior to baselines and related works in dealing with the NER task for dairy cow diseases.

## 2. Materials and Methods

### 2.1. Dataset

Relevant knowledge of dairy cow diseases commonly exists in the professional literature as well as on online websites, and some exists in the real-world medical records of sick dairy cows recorded by veterinarians; to ensure the robustness of our model, we collected texts about dairy cow diseases from these three types of data sources.

**The professional literature.** We used four professional books published by Chinese scholars, including “奶牛常见疾病诊疗手册 (*Handbook of Diagnosis and Treatment of Common Dairy Cow Diseases*)” [32], “奶牛疾病诊疗大全 (*Comprehensive Diagnosis and Treatment of Dairy Cow Diseases*)” [33], “奶牛疾病诊疗技术 (*Diagnosis and Treatment Technologies of Dairy Cow Diseases*)” [34], and “奶牛疾病诊治彩色图谱 (*Diagnosis and Treatment Graph of Dairy Cow Diseases*)” [35], and the Chinese version of an English professional book, “奶牛疾病学 (*Rebhun’s Diseases of Dairy Cattle*)” [36]. They are all authoritative books in the field of dairy cow diseases, which can offer reliable knowledge of dairy cow diseases.

**Online websites.** Meanwhile, we used web crawler technology to collect texts about dairy cow diseases from two websites, including Baidu Baike (baike.baidu.com (accessed on 16 August 2023)) [37] and a knowledge service website named the Agricultural Knowledge Service System (http://agri.nais.net.cn (accessed on 21 September 2023)) [38] built by the Agricultural Information Institute of the Chinese Academy of Agricultural Sciences (CAAS), which can offer open and shared knowledge of dairy cow diseases.

**Medical records.** Additionally, we collected the real-world medical records of 832 dairy cows from 13 large-scale livestock farms in Heilongjiang, China. These records were maintained by the Dairy Association of Heilongjiang Province (DAHLJ), which is one of the partners of our team. Each record contained diagnosis results, clinical symptoms, laboratory examinations, medicine applications, and disposal measures, which could offer real-world knowledge of dairy cow diseases.

**Figure 1:**
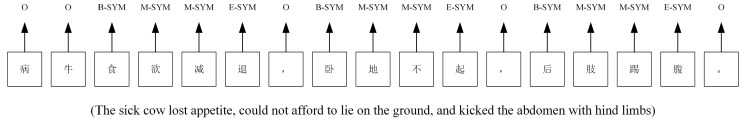
An illustration of the BMESO annotation method.

For the collected data of dairy cow diseases, we first deleted non-textual parts such as images and charts, removed irrelevant characters, special symbols, punctuation marks, and HTML tags, and revised wrongly written characters. After data cleaning, we obtained a high-quality dataset containing 16,472 sentences with an average sentence length of 127 characters. Then, we applied the BMESO [39] method to annotate each sentence, shown in Figure 1, where B, M, and E, respectively, represent the beginning, middle, and end positions of an entity, S represents an entity with a single character, and O represents a character which is not an entity. For example, “病 (disease)” and “牛 (cow)” were non-entity characters, so we marked them with the label “O”; “食欲减退 (decreased appetite)” was a symptom entity, so we used the label “B-SYM” to mark its first character, used the label “M-SYM” to mark its middle character, and used the label “E-SYM” to mark its end character, in which “SYM” corresponded to the entity type of symptom. This annotation was helpful in capturing entity boundaries. To ensure the consistency and reliability of data annotation, a small portion of the corpus was first extracted and annotated together by all of the annotators so as to develop the first version of annotation rules. Then, the corpus was annotated in batches, and each annotator was assigned a quantitative annotation task. After each batch of annotation tasks was completed, annotators checked the annotation content with each other and discussed the inconsistent annotation content until they reached a consensus.

**Figure 2:**
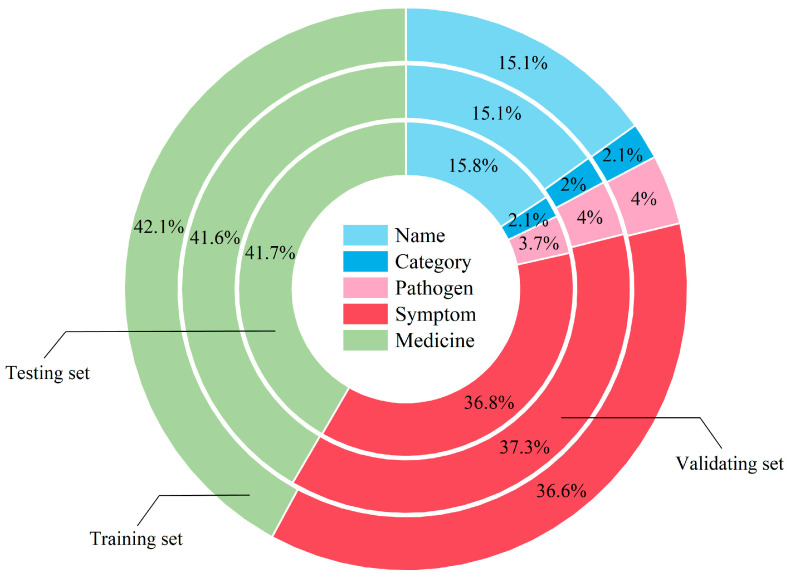
The distribution of each entity type in our dairy cow disease dataset. The percentages in the figure represent the proportion of each type of entity to the total number of entities in the training, validation, or testing sets. For example, the proportions of disease name, category, pathogen, symptom, and medicine in the training set are 15.1%, 2.1%, 4%, 36.6%, and 42.1%, respectively.

We mainly focused on five types of entities, including the disease name, symptom, medicine, pathogen, and disease category, which are directly relevant to disease diagnosis. Through data annotation, we obtained a labeled dataset containing 16,472 sentences, with a total of 39,117 named entities in the dataset. Then, we divided it into a training set, a validation set, and a testing set in a ratio of 6:2:2. The training set, validation set, and testing set contained 9883, 3295, and 3294 sentences with 22,786, 8280, and 8051 entities, respectively. The data distribution for each entity type is illustrated as Figure 2.

### 2.2. Model

**Figure 3:**
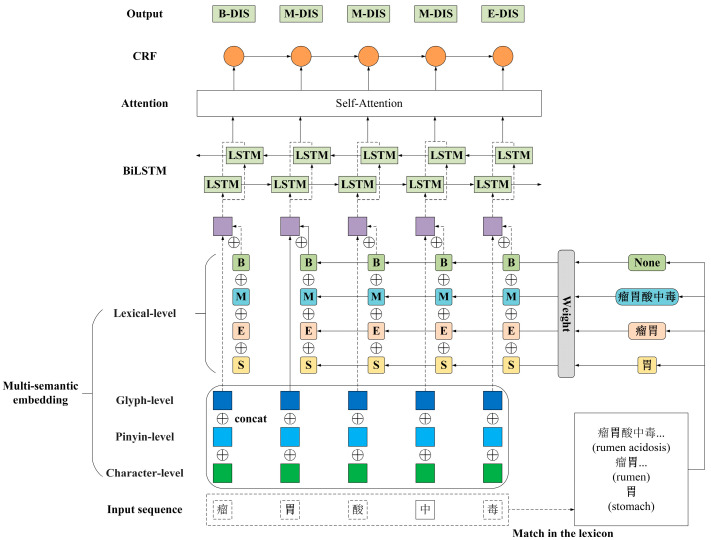
The overall architecture of our model. B, M, E, and S denote the four word set vectors obtained by compressing the four word sets matched by the Chinese characters in the lexicon.

The overall architecture of our proposed model is shown as Figure 3. Our model is composed of four modules called the multi-semantic embedding layer, Bi-LSTM layer, self-attention layer, and CRF layer. First (Section 2.2.1), the multi-semantic embedding layer obtained the character-level, glyph-level, lexical-level, and pinyin-level representations from a text sequence about dairy cow diseases. Then (Section 2.2.2 and Section 2.2.3), these multi-level representations were concatenated and input into the Bi-LSTM encoder integrated with a self-attention mechanism. This mechanism enabled the encoder to focus on important features and learn long-range dependencies, thereby extracting relevant features. Finally (Section 2.2.4), the extracted features were further input into the CRF decoder to obtain the globally optimal label sequence corresponding to the text sequence.

#### 2.2.1. Multi-Semantic Embedding Layer

The multi-semantic embedding layer mapped the text sequence of dairy cow diseases into a lower-dimensional vector space, obtaining vector representations of the text characters. For an input text sequence of dairy cow diseases $X={\{c}_{1},c_{2},c_{3},\cdots ,c_{n}\}$, where n denotes the sequence length, the multi-semantic embedding layer converts this sequence into its embedded representation $E={(e}_{1},e_{2},\cdots ,e_{n})$, with $e_{i}=e_{i}^{c}⨁e_{i}^{g}⨁e_{i}^{s}⨁e_{i}^{p}$, where $e_{i}^{c}$, $e_{i}^{g}$, $e_{i}^{s}$, and $e_{i}^{p}$ denote the vectors of the character feature, glyph feature, lexical feature, and pinyin feature, respectively. By concatenating these feature vectors, we obtained multi-level semantic representations of Chinese characters in the dairy cow disease corpus.

**Character-level Embedding.** Word2vec [40] has been commonly used for converting characters into vector representations. It is capable of capturing contextual semantic information between words by training on large-scale corpora, thus obtaining semantic vector representations that closely reflect the real distribution of target characters and words. Therefore, we employed word2vec from the gensim library (version 4.2.0) for pre-training on a self-constructed dairy cow disease corpus, with fine-tuning during training. For $X={\{c}_{1},c_{2},c_{3},\cdots ,c_{n}\}$, where n represents the sequence length, we used the word2vec model to obtain its vector sequence $E^{c}=(e_{1}^{c},e_{2}^{c},\cdots ,e_{n}^{c})$, where $e_{i}^{c}$ denotes the character-level feature vector corresponding to $c_{i}$.

**Pinyin-level Embedding.** Pinyin represents the formal pronunciation of Chinese characters. Each character’s pronunciation is linked to its specific meanings, providing auxiliary information for character-level semantic interpretation. For example, the Chinese character “好 (good)” is a polyphonic character. When pronounced as “hǎo”, it typically conveys a positive evaluation, and when pronounced as “hào”, it denotes a strong interest or hobby. Due to the diversity of data sources, an entity of dairy cow diseases referring to the same meaning might be expressed differently in various contexts, sometimes due to homophones. For example, “瘤胃臌气 (Rumen distension)” and “瘤胃鼓气 (Rumen distension)” differ by only one character, where “臌 (swell)” and “鼓 (bulge)” are homophones, and they share the same pinyin sequence {liu, wei, gu, qi}, which can be used as a basis for regarding them as the same entity. To address homophone issues, we incorporated pinyin into character representations to enhance the performance of named entity recognition for dairy cow diseases.

**Figure 4:**
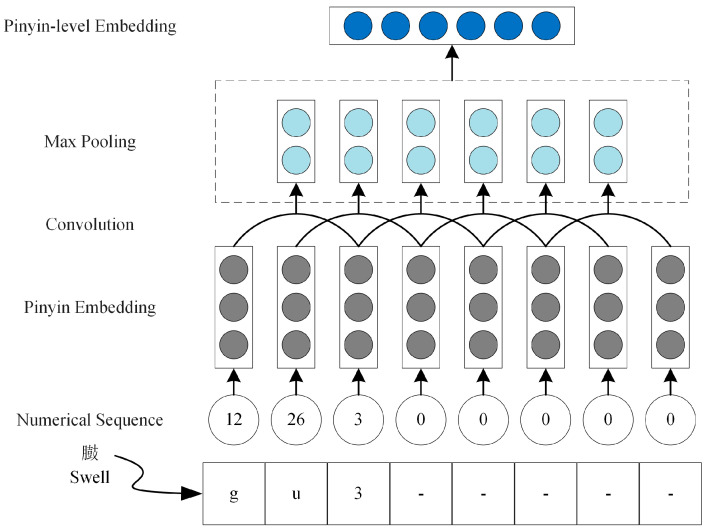
The architecture of pinyin-level embedding. “gu3” in the figure represents the pinyin sequence corresponding to the Chinese character “臌 (swell)”.

The architecture of pinyin-level embedding is shown as Figure 4. An open-source Pypinyin package (version 0.7.2) was utilized to generate pinyin sequences for Chinese characters, which is a system that combines machine learning models and rules to infer the pinyin of a given character according to its context. Each character’s tone was represented by the digits 0 to 4 and appended to its pinyin sequence. Referring to [41], a mapping table was used to convert the pinyin sequence into an 8-digit numerical sequence. Sequences shorter than 8 digits were padded with zeros. For example, the pinyin sequence of the character “臌 (swell)” was {gu3}, which was encoded as {12, 26, 3, 0, 0, 0, 0, 0}. The numerical sequence was then processed through a 2 × 2 convolutional layer and a max pooling layer to generate the pinyin vector representation. For $X={\{c}_{1},c_{2},c_{3},\cdots ,c_{n}\}$, we obtained $E^{p}=(e_{1}^{p},e_{2}^{p},\cdots ,e_{n}^{p})$ by the pinyin-level embedding, where $e_{i}^{p}$ represents the pinyin-level feature vector corresponding to $c_{i}$.

**Glyph-level Embedding.** Chinese characters are hieroglyphs; the structure of a character contains its semantic information, which can be useful for named entity recognition. For example, the Chinese character “岩 (cliff)” is a vertically structured character consisting of two parts, namely “山 (mountain)” and “石 (stone)”. It is related to mountains with rocks and often appears in geological and mineralogical texts. In dairy cow disease text sequences, there are many hieroglyphs referring to disease or the location of disease. For example, in disease name entities, such as “结核病 (tuberculosis)” and “口蹄疫 (foot-and-mouth disease)”, both characters “病 (disease)” and “疫 (epidemic)” share the same radical “疒” which commonly indicates diseases. In symptom entities, such as “眼睑肿胀 (eyelid swelling)”, the characters “肿 (swell)” and “胀 (inflate)” share the radical “月 (round)”, which usually indicates body tissues. Differently from the methods in previous studies that split Chinese characters into radical sequences and using a one-dimensional convolutional neural network (1D CNN) to extract glyph features from the sequences, we extracted glyph features directly from the images of Chinese character sequences, thus capturing more comprehensive glyph structure information.

**Figure 5:**
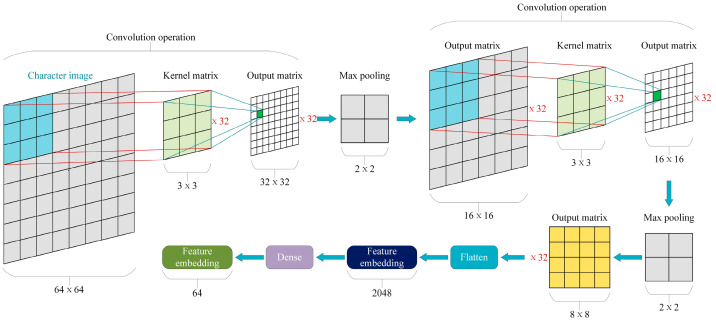
The implementation of image feature embedding. The “64 × 64” square represents the feature matrix of Chinese character images, the “3 × 3” square represents a convolution kernel, the “32 × 32” square represents the output matrix obtained through convolution processing, and the “2 × 2” square represents the max pooling layer. In addition, the “8 × 8” square represents the output of the second convolutional layer.

We employed the GLYNN [42], which was originally designed for image classification tasks, to extract features from Chinese character images. The implementation details are illustrated in Figure 5. Firstly, the sentence sequence of dairy cow diseases was converted into Chinese character images, with each image sized 64 × 64 pixels and one channel as the images were achromatic. In order to simulate the image representation of non-Chinese characters, we adopted a random matrix sized 64 × 64 with element values between 0 and 1. Then, the Chinese character image sequence was processed through two convolutional layers. Both layers utilized 3 × 3 convolution kernels, with 32 output channels. In the first convolutional layer, the sigmoid activation function was applied, with the stride set to 2, enabling non-linear transformations and dimensionality reduction in features. In the second convolutional layer, the ReLU activation function was applied to accelerate the training process and mitigate the vanishing gradient problem, with the stride set to 1. After each convolutional layer, a batch normalization layer was employed to speed up training and improve the model stability, followed by a max pooling layer to reduce the network’s computational complexity and spatial dimensions. Additionally, two dropout layers were included to prevent overfitting. The multidimensional vector was then converted into a one-dimensional vector through the Flatten operation for subsequent processing. Following this, the vector size was further reduced through a fully connected layer. Finally, a feature vector with 64 dimensions was obtained. For $X={\{c}_{1},c_{2},c_{3},\cdots ,c_{n}\}$, we obtained $E^{g}=(e_{1}^{g},e_{2}^{g},\cdots ,e_{n}^{g})$ by glyph-level embedding, where $e_{i}^{g}$ represents the glyph-level feature vector corresponding to $c_{i}$.

**Lexical-level Embedding.** In English corpora, the word is the smallest unit; each word can express its meaning independently, and different words are clearly separated by spaces. Meanwhile, in Chinese corpora, the Chinese character is the smallest unit; words that are composed of at least two characters can express more semantic information than a single character, and there is a lack of explicit boundaries between adjacent characters, which increases the complexity of Chinese named entity recognition. Traditional Chinese NER methods based on word segmentation have the problem of error propagation caused by incorrect word segmentation, while methods based on character-level information are not capable of utilizing entity boundary information, thereby losing substantial lexical information crucial for entity recognition. To this end, we referenced the approach of SoftLexicon [22] to introduce external lexical information so as to enhance the recognition performance of dairy cow disease entities, especially complex entities composed of special terms.

**Figure 6:**
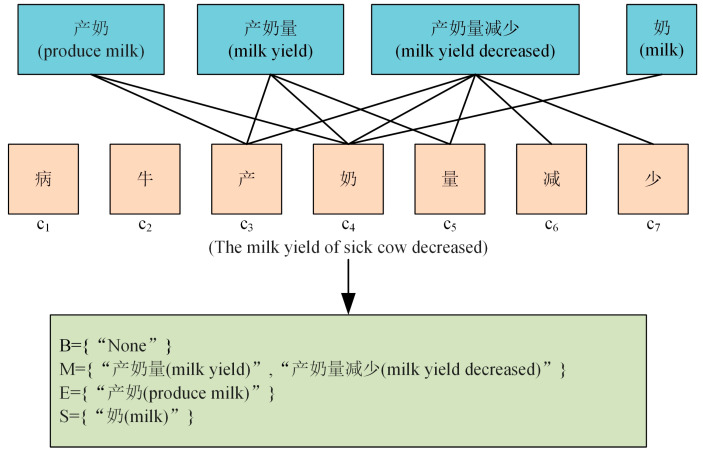
The illustration of lexical-level embedding. The words in the blue box are from our pre-trained lexicon. The words in the orange box are from a disease text sequence. B, M, E, and S in the green box represent the set that matches the Chinese character “奶 (milk)” in our lexicon.

We labeled our dataset by the BMESO annotation method. For each character $c_{i}$ in the input sequence $X={\{c}_{1},c_{2},c_{3},\cdots ,c_{n}\}$, we obtained $B\left(c_{i}\right)$, $M\left(c_{i}\right)$, $E\left(c_{i}\right)$, and $S\left(c_{i}\right)$ by matching a pre-trained lexicon, where $B\left(c_{i}\right)$ represents the set of words that started with the character $c_{i}$ in the lexicon, $M\left(c_{i}\right)$ denotes the set of words where $c_{i}$ was in the middle of them in the lexicon, $E\left(c_{i}\right)$ represents the set of words that ended with $c_{i}$ in the lexicon, and $S\left(c_{i}\right)$ denotes the character $c_{i}$ itself. If no matching words were found in the lexicon, “None” would be added to the set. As shown in Figure 6, for the input sequence ”病牛产奶量减少 (The milk yield of sick cow decreased)”, using c_4_ as an example, the matching results from the lexicon were as follows: $B\left(c_{4}\right)$ = {“None”}; $M\left(c_{4}\right)$ = {“产奶量 (milk yield)”, “产奶量减少 (milk yield decreased)”}; $E\left(c_{4}\right)$ = {“产奶 (produce milk)”}; and $S\left(c_{4}\right)$ = {“奶 (milk)”}. For each character, we obtained four word sets corresponding to it and compressed each set into a fixed-dimensional word set vector according to its word frequency as follows:

(1) $$v^{s}\left(K\right)=\frac{4}{Z}\sum_{w\in K}z\left(w\right)e^{w}\left(w\right)$$

(2) $$Z=\sum_{w\in B\cup M\cup E\cup S}z\left(w\right)$$

where K∈{B,M,E,S} and z(w) denotes the frequency of the word w in the lexicon, which reflects the likelihood of w being a correct word boundary. $e^{w}(w)$ represents the word embedding vector obtained from pre-trained word vectors, and $v^{s}(K)$ denotes the word set vector of the K-word set. Z is the total number of words in the four word sets.

Finally, the word set vectors $v^{s}(B)$, $v^{s}(M)$, $v^{s}(E)$**,** and $v^{s}(S)$ corresponding to each character $c_{i}$ were concatenated to form the lexical feature vector $e^{s}$, which is denoted as follows:

(3) $$e^{s}\left(B,M,E,S\right)={[v}^{s}\left(B\right);v^{s}\left(M\right);v^{s}\left(E\right);v^{s}\left(S\right)]$$

For $X={\{c}_{1},c_{2},c_{3},\cdots ,c_{n}\}$, we obtained $E^{s}=(e_{1}^{s},e_{2}^{s},\cdots ,e_{n}^{s})$ by lexical-level embedding, where $e_{i}^{s}$ represents the lexical feature vector corresponding to character $c_{i}$.

#### 2.2.2. Bi-LSTM Layer

In order to accurately identify named entities for dairy cow diseases, we employed a Bi-LSTM network to represent the text in both forward and backward directions so as to fully extract contextual semantic features. The multi-level representation vector $e_{t}={(e}_{1},e_{2},\cdots ,e_{n})$ obtained from the multi-semantic embedding layer was fed into the network step by step, and the hidden representation of $e_{t}$ was computed by

(4) $$h_{t}=\left[\vec{h_{t}};\overset{\leftarrow }{h_{t}}\right],t=1,\cdots ,n$$

(5) $$\vec{h_{t}}=\vec{LSTM}\left(e_{t},\vec{h_{t-1}}\right)$$

(6) $$\overset{\leftarrow }{h_{t}}=\overset{\leftarrow }{LSTM}\left(e_{t},\overset{\leftarrow }{h_{t-1}}\right)$$

where $h_{t}$ represents the hidden state in the time step t, with $\vec{LSTM}$ and $\overset{\leftarrow }{LSTM}$ representing the forward and backward sub-LSTM networks, respectively.

#### 2.2.3. Self-Attention Layer

In the text sequences of dairy cow diseases, only a few relevant characters are crucial for NER and should be allocated a large weight, while other unimportant characters should be given a small weight. However, Bi-LSTM cannot achieve such weight allocation effectively. Additionally, as the distance between characters increases, the ability of Bi-LSTM to capture long-distance dependencies decreases. To deal with these issues, we employed a self-attention mechanism to enable our model to focus on important characters and capture long-distance dependencies in long sentences.

**Figure 7:**
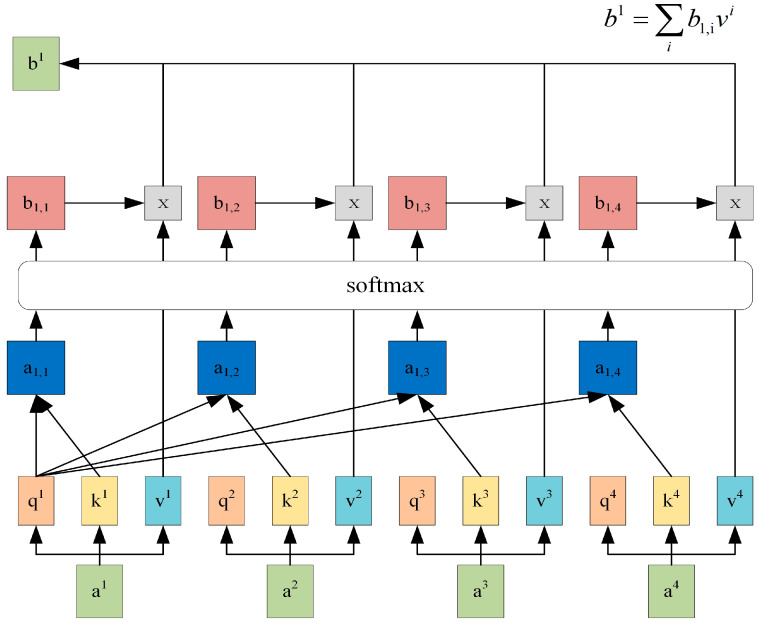
The calculation process of a^1^’s self-attention.

As shown in Figure 7, the calculation process of self-attention is illustrated here using $a^{1}$ as an example. (1) Given the query vector $q^{1}\subseteq Q={\left[q^{1},q^{2},\ldots ,q^{n}\right]}^{T}$ and the key vector $k^{i}\subseteq K={\left[k^{1},k^{2},\ldots ,k^{n}\right]}^{T}$, the similarity score between $a^{1}$ and each key vector $k^{i}$ was obtained by calculating the dot product of $q^{1}$ with $k^{i}$. (2) The similarity score was then divided by $\sqrt{d_{k}}$ to achieve a more stable gradient. (3) The softmax function was applied to compute the weight coefficient $b_{1,i}$. (4) Finally, each value vector $v^{i}\subseteq V={\left[v^{1},v^{2},\ldots ,v^{n}\right]}^{T}$ was weighted by the coefficient $b_{1,i}$ and summed to obtain the attention output. The scaled dot-product attention was computed by

(7) $$Attention\left(Q,K,V\right)=softmax\left(\frac{QK^{T}}{\sqrt{d_{k}}}\right)V$$

where Q, K, and $V\in R^{n\times 2l}$ represent the query matrix, key matrix, and value matrix, respectively, and l denotes the hidden dimension of the LSTM cell. $d_{k}$ is the vector dimension of K. We set Q=K=V=H, where H represents the output matrix of the Bi-LSTM.

To extract text features from multiple perspectives, we employed a multi-head self-attention mechanism, which adjusted the weights of the Bi-LSTM output matrix and performed scaled dot-product attention with linear transformations on r parallel heads, each focusing on different parts, which is defined as follows:

(8) $$MultiHead\left(Q,K,V\right)=\left[{head}_{1};\cdots ;{head}_{r}\right]W^{O}$$

(9) $${head}_{i}=Attention\left(QW_{i}^{Q},KW_{i}^{K},VW_{i}^{V}\right)$$

where [;] represents concatenation, r is the number of heads, and $W^{O}\in R^{2l\times 2l}$ is the trainable weights. $W_{i}^{Q}$, $W_{i}^{K}$, and $W_{i}^{V}\in R^{2l\times d}$ are trainable weights and d=2l/r.

#### 2.2.4. CRF Layer

Finally, we employed the CRF [43] layer to add a constraint relation to the final predicted label to ensure its rationality. Given the input sequence $X={\{x}_{1},x_{2},x_{3},\cdots ,x_{n}\}$, we assumed that the predicted label sequence was $Y={\{y}_{1},y_{2},y_{3},\cdots ,y_{n}\}$. We took the output sequence A of the self-attention mechanism as the input of the CRF, and the score of the label sequence y was computed by

(10) $$score\left(X,y\right)=\sum_{i=1}^{n}A_{i,y_{i}}+T_{y_{i-1},y_{i}}$$

where T represents the transfer matrix, $T_{y_{i-1},y_{i}}$ represents the transition probability from the labels $y_{i-1}$ to $y_{i}$, and $A_{i,y_{i}}$ represents the score for assigning the label $y_{i}$ to the i-th character in the sequence.

We used the softmax function to calculate the conditional probability P(y|X) of the label sequence y, which is represented as follows:

(11) $$P\left(y|X\right)=Softmax\left(score\left(X,y\right)\right)=\frac{e^{score\left(X,y\right)}}{\sum_{\tilde{y}\in Y_{x}}e^{score\left(X,\tilde{y}\right)}}$$

where $Y_{x}$ represents the set of all possible label sequences; the conditional probability corresponding to the globally optimal label sequence is the highest.

During the decoding process, we used the Viterbi algorithm [44] from the pytorch-crf library (version 0.7.2) to predict the optimal label sequence $y^{*}$, which is represented as follows:

(12) $$y^{*}=arg\;\underset{y\in Y_{x}}{\max}\left(score\left(X,y\right)\right)$$

### 2.3. Experimental Setup

#### 2.3.1. Parameters

We utilized PyTorch to implement our proposed PGLA-DCNER model on RTX A5000 GPU. The versions of Python and PyTorch were 3.8.0 and 1.12.0, respectively. The hidden-layer dimension of the single-layer Bi-LSTM was set to 300, and the maximum number of iterations was set to 80. The specific parameters of our model are illustrated in Table 1.

Meanwhile, we employed the word2vec model to pre-train a lexicon on a large-scale dairy cow disease corpus, which was useful in capturing lexical features of dairy cow disease texts. Finally, we evaluated the model performance using precision (P), recall (R), and the F1 score (F1) and used the limitation that an entity was correctly recognized only when its boundaries (namely the leftmost character and the rightmost character of a Chinese word) and type were both accurately recognized.

#### 2.3.2. Models

To evaluate the effectiveness of our proposed model for the NER task in the field of dairy cow disease, we conducted experiments with 18 models, which are summarized in Table 2. We compared four types of models: baseline models, our model, related works, and ablation models. SoftLexicon models with a CNN, a Transformer, and Bi-LSTM as encoders were regarded as baselines, which did not take into account pinyin-level and glyph-level features as well as attention mechanisms. To determine whether each component of our model contributed to the results, we conducted 6 ablation experiments; for example, “+pinyin” is a model that uses pinyin-level features based on the best baseline model, while “+pinyin+glyph” is a model that uses both pinyin-level and glyph-level features based on the best baseline model. To validate the superiority of our model, we further compared our model with 8 models from related works.

## 3. Results

### 3.1. Recognition Performances of Different Models

To evaluate the named entity recognition performance of our proposed PGLA-DCNER model, we first compared our model with baseline models and other mainstream NER models on a dataset of dairy cow diseases. For baseline models, we compared the performance of the SoftLexicon model [22] using different encoders: a CNN, a Transformer, and Bi-LSTM. For related works, we compared two types of models, namely character-based models and ensemble models. Character-based models included Bi-LSTM-CRF [45], Bi-LSTM-Attention-CRF [46], BERT-Bi-LSTM-CRF [47], and RoBERTa-Bi-LSTM-CRF [48], while ensemble models incorporating character, lexical, or glyph features included WC-LSTM [49], LR-CNN [50], FLAT [23], and MECT [30]. The evaluation results of our model and those from related works are listed in Table 3.

#### 3.1.1. Comparison with Baselines

Our model outperformed the baselines which did not all take into account pinyin-level and glyph-level features as well as attention mechanisms. In terms of the F1 score, the performance of our model was 92.18%, up by 2.83% compared with the best baseline, SoftLexicon (Bi-LSTM), which used Bi-LSTM as an encoder. This suggests that the various components we introduced were indeed beneficial for the NER task in this article.

We further discuss which kind of encoder was more suitable for the dairy cow disease corpus among the baseline models. As shown in Table 3, the Bi-LSTM encoder performed the best among the three baselines in terms of the F1 score, which improved by 11.35% and 4.29% compared with that of the CNN encoder and Transformer encoder. In addition, the F1 score of the Transformer encoder achieved an improvement of 6.77% compared to that of the CNN encoder.

#### 3.1.2. Comparison with Related Works

Our model also outperformed the models from related works, most of which only used one or two types of features and none of which used pinyin-level features, while Bi-LSTM-Attention-CRF employed an attention mechanism. The performance of our model improved by 2.63% and 1.80% in the F1 score compared with the best character-based model, BERT-Bi-LSTM-CRF, and the best ensemble model, MECT, respectively.

Specifically, for character-based models, Bi-LSTM-CRF, taking into account character-level vectors and employing Bi-LSTM to extract context information, achieved an F1 score of 87.71%. Bi-LSTM-Attention-CRF had an improvement of 0.59% over Bi-LSTM-CRF by introducing an attention mechanism, which made it able to focus on proper content. Differently from Bi-LSTM-CRF, BERT-Bi-LSTM-CRF employed a pre-trained BERT model instead of word2vec for text representation, which improved the F1 score by 2.41%. RoBERTa-Bi-LSTM-CRF generated character vectors using a pre-trained RoBERTa model, which performed almost the same as BERT-Bi-LSTM-CRF but better than Bi-LSTM-CRF.

Meanwhile, for ensemble models, WC-LSTM, incorporating lexical information into the beginning or end characters of words which mitigated the impact of word segmentation errors, improved the F1 score by 1.22% compared with Bi-LSTM-CRF. LR-CNN integrated lexical information on the basis of a CNN using the Rethinking mechanism, which improved the F1 score by 1.21% compared to Bi-LSTM-CRF and performed almost the same as WC-LSTM. Although WC-LSTM and LR-CNN, which incorporate external lexical information, outperformed Bi-LSTM-CRF, their performances were still inferior to that of BERT-Bi-LSTM-CRF and RoBERTa-Bi-LSTM-CRF which utilized pre-trained language models for text representation. FLAT, using a position encoding method to introduce external lexical information, achieved an F1 score of 90.54%, which was superior to those of the above models that introduced lexical information. MECT further incorporated the structural information of Chinese characters on the basis of FLAT, which achieved a slight improvement compared with FLAT.

### 3.2. Ablation Study

Next, we investigated how each component contributed to our model. Since our proposed PGLA-DCNER model is an improved model based on SoftLexicon which already takes into account character-level and lexical-level features, we mainly conducted ablated experiments to see the contributions of pinyin-level embedding, glyph-level embedding, and attention modules, and the experimental results are shown in Table 4. According to the results, we can see that the two embedding methods and the attention mechanism all had positive influences on our model, among which the pinyin-level embedding had the greatest contribution with an improvement of 1.20% compared with baseline model. After introducing both pinyin-level and glyph-level embeddings, the recognition performance was further improved by 0.37% and 1.17% compared with that of pinyin-level embedding and glyph-level embedding, respectively. When all components were introduced in our model, the F1 score was improved by 2.83% compared to that of the baseline SoftLexicon.

#### 3.2.1. Pinyin-Level Embedding

As shown in Table 4, in terms of the F1 score, +pinyin using pinyin-level features performed better than the +glyph model, with an increase of 1.20% compared with the baseline model. In addition, our PGLA-DCNER model had an improvement of 1.59% compared with +glyph+attention model which did not use pinyin-level embedding, and the +pinyin+glyph model achieved a score 1.17% higher than +glyph model while the +pinyin+attention model achieved a score 0.61% higher than the +attention model.

#### 3.2.2. Glyph-Level Embedding

It can be seen from Table 4 that +glyph using glyph-level features had an improvement of 0.41% in the F1 score compared with the baseline model. Compared to the +pinyin+attention model, our PGLA-DCNER model improved by 1.27%, owing to its glyph-level embedding. In addition, the +pinyin+glyph model achieved a score 0.37% higher than the +pinyin model while the +glyph+attention model achieved a score 0.298% higher than the + attention model.

To further investigate the effectiveness of PGLA-DCNER in capturing glyph features, we conducted additional experiments and compared three models, Radical_CNN, Tianzige_CNN, and GLYNN, with GLYNN mentioned in Section 2.2.1. For Radical_CNN, we first split the Chinese characters into radical sequences and subsequently processed these sequences using a convolutional neural network with a convolutional kernel size of 3×3 to obtain radical features. For Tianzige_CNN, we first converted the Chinese characters into corresponding images and then employed a convolutional layer with a kernel size of 5 × 5 to capture the lower-level glyph features. The output of the convolutional layer was further processed using a max pooling layer with a kernel size of 4 × 4 to reduce the resolution from 8 × 8 to 2 × 2, which reflected the arrangement of the radicals of Chinese characters. Finally, we used group convolution [51] to map the Tianzige grid to the final output. The experimental results are shown in Table 5. We can see that there was no significant difference between Radical_CNN and Tianzige_CNN in terms of the F1 score; although Radical_CNN performed better, it still did not achieve our expected result. However, GLYNN achieved an improvement of 0.49% and 0.90% compared with Radical_CNN and Tianzige_CNN, respectively. This implies that the glyph feature extraction method proposed in our PGLA-DCNER model was able to exploit the morphological features embedded in Chinese characters more efficiently, thus improving the performance of our proposed model in recognizing named entities in the Chinese dairy cow disease corpus.

#### 3.2.3. Self-Attention Mechanism

In terms of the F1 score, our PGLA-DCNER model had an improvement of 1.23% compared with the +pinyin+glyph model which did not use an attention mechanism. Meanwhile, the + attention model improved by 0.93% compared with the baseline model, and +pinyin+attention achieved a score 0.37% higher than the +pinyin model, and the +glyph+attention model achieved a score 0.81% higher than the +glyph model.

### 3.3. Performance on Different Entity Types

**Figure 8:**
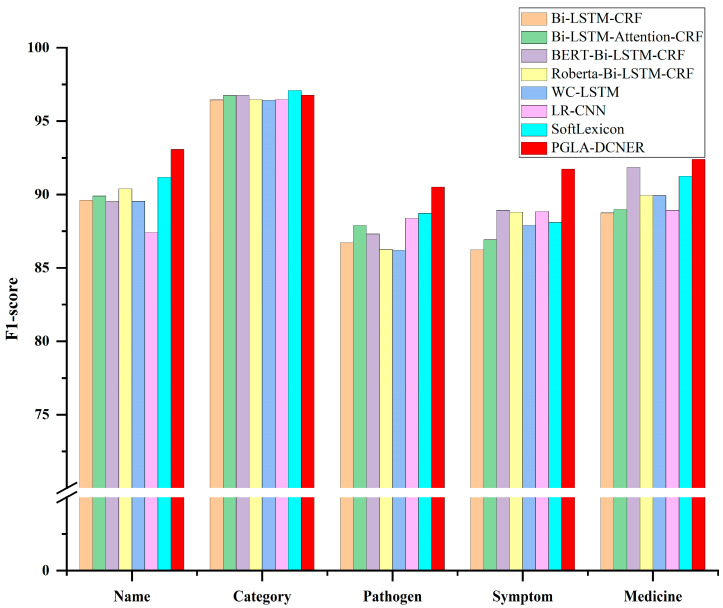
Performances of different models with different entity types.

To evaluate the performance of our proposed PGLA-DCNER model in recognizing different types of named entities, we calculated the F1 scores of different models for each entity type. The experimental results are shown as Figure 8. In terms of the F1 score, Bi-LSTM-CRF achieved 89.61%, 96.44%, 86.71%, 86.23%, and 88.75% for the disease name, category, pathogen, symptom, and medicine, respectively. Benefiting from the boundary information provided by the external lexicon employed in SoftLexicon, the F1 scores for each entity type improved by 1.80%, 0.67%, 2.30%, 2.20%, and 2.80%, respectively, compared with those of Bi-LSTM-CRF. Based on SoftLexicon, our proposed PGLA-DCNER model, which further took into account pinyin-level and glyph-level features and the self-attention mechanism, achieved optimal performance with each entity type except the disease category, and the F1 scores increased by 2.10%, 2.00%, 4.10%, and 1.20% for the disease name, pathogen, symptom, and medicine and decreased by 0.30% in the disease category.

### 3.4. Computation Performances of Different Models

In order to investigate the training efficiency and resource consumption of our proposed PGLA-DCNER model, we compared it with other models considering training time, inference speed, and memory usage. The experimental results are shown in Table 6. The results indicate that the proposed model took 130.41 s for one iteration, which was longer than the time that the baseline model SoftLexicon took. This may have been due to the introduction of multi-semantic features and attention mechanisms, which increased the complexity of the model and required more time for convergence. In addition, the training time of PGLA-DCNER was much longer than that of the Transformer architectures such as FLAT and MECT, which may have been limited by the inability of the models with Bi-LSTM architectures to fully utilize GPUs for parallel training. In terms of inference speed, there was a situation similar to that of the training time, which may still have been due to the high complexity of the model and the limitations of its own architecture. In terms of memory usage, PGLA-DCNER’s was higher than that of the other compared models, which may have been due to the introduction of glyph, pinyin, and lexical features that required a large amount of locally stored resources such as images and mapping tables, which increased memory consumption.

## 4. Discussion

### 4.1. Performance Analysis of Baselines and Related Works

In comparison with the baselines, we found that the CNN was not suitable for our task, mainly because its receptive field was limited, making it difficult to effectively capture long-distance dependencies. This limitation of the CNN was previously noted in [23]. Although the Transformer has advantages in capturing long-distance dependencies, its performance on our dataset was still slightly lower than that of Bi-LSTM. This may be because the Transformer’s ability to extract contextual features and capture long-range dependencies is not as strong as Bi-LSTM’s. Therefore, we ultimately chose Bi-LSTM as the encoder for our model. In comparison with related works, our model performed significantly better than two representative models, BERT-Bi-LSTM-CRF and MECT. This is mainly attributed to our model’s ability to capture the multi-level features of Chinese characters, thereby more comprehensively representing their semantic information. Furthermore, by introducing attention mechanisms, our model was able to better learn long-range dependencies between Chinese characters. Previous studies [28,29] have demonstrated the importance of extracting multi-semantic features and incorporating attention mechanisms. Additionally, as highlighted in [26], the word2vec word embedding method has limitations in handling polysemy, making large language models more advantageous for generating word embeddings. Although models such as WC-LSTM and LR-CNN have achieved good recognition performance by introducing external lexical information, their reliance on word2vec for word embeddings limits their performance compared to BERT-Bi-LSTM-CRF, which use large language models for word embeddings. Moreover, in our experiments, MECT was only slightly superior to FLAT. This result may be related to the Chinese character splitting dictionary used during the reproduction of the MECT model. Unlike the original paper, we used an alternative dictionary, which divided Chinese characters more roughly compared to the more thorough division in the original dictionary. This difference may explain the lower performance of our reproduced model compared to the results reported in the original paper.

Finally, comparative experiments on model training efficiency and other aspects showed that the proposed model did not have significant advantages in training time, inference speed, and memory usage. There is still considerable room for improvement in these areas. In future work, we will focus on optimizing the computational efficiency and resource utilization of the model to address these limitations.

### 4.2. Effects of Each Component of Our Model

The results of the ablation experiment indicate that the introduced components had a positive impact on the performance of the model. The specific reasons are as follows: Firstly, the introduction of lexical features enhanced the model’s ability to recognize entity boundaries, while pinyin features effectively overcame the problems caused by homophones. Secondly, capturing glyph features from the Chinese character images of the dairy cow disease corpus helped the model better recognize the overall structure of Chinese characters. Additionally, the introduction of the attention mechanism enabled the model to focus on important information for identifying entities in the corpus, thereby improving overall performance. Finally, we found that the combination of multiple features significantly outperformed the use of a single feature. This indicates that there was complementarity among different features, which collectively enhanced the performance of our model. Unlike previous studies that have relied on only one or two features, our model introduced a richer set of features, further demonstrating its advantages.

### 4.3. Performance Analysis on Different Entity Types

The experimental results show that, compared with other models, our model had a significant advantage in extracting named entities for almost all categories. However, it was less effective in extracting certain types of entities, such as disease category entities. This was primarily because the boundaries of disease category entities were relatively consistent, with most entities ending with the Chinese character “病 (disease)”. However, some disease name entities also ended with “病 (disease)”, and the introduction of glyph features may have confused these two types of entities, leading to a decrease in the recognition performance of disease category entities. Additionally, we can see that almost all models performed relatively worse with symptom and pathogen entities compared to other types. For symptom entities, although their quantity was large, the length of each symptom was longer, and their semantic expressions were highly diverse, which increased the difficulty of identifying such entities. For pathogen entities, their low frequency in the dataset made it challenging for our model to fully learn their semantic information. In contrast, the expressions of disease name, category, and medicine entities were relatively fixed, and they appeared more frequently in the dataset. Moreover, they often had obvious boundary features, such as “病 (disease)”, “炎 (inflammation)”, “素 (element)”, and “液 (liquid)”. These characteristics enabled our model to learn more semantic information about them, thus resulting in better performance.

In conclusion, our model was proved to be suitable and effective for named entity recognition for a dairy cow disease corpus. Our model is also applicable for other livestock diseases or crop pests and diseases, since the characteristics of their disease texts are similar. For example, in the agricultural field, the crop cultivar entity “GS豫豆8号 (GS Yudou 8)” is a mix of numbers, letters, and Chinese characters, while “瘟 (acute communicable disease)” and “病 (disease)” in the disease entity “稻瘟病 (rice blast)” have the same radical “疒”. The recognition of the two types of entities mentioned above can benefit from the introduced lexical features and glyph features, respectively. In addition, our model can be properly integrated into farm management systems as a sub-module, and combined with a relation extraction sub-module, it is feasible to automatically build a professional knowledge graph of dairy cow diseases. However, it is noted that our model does not currently have a significant advantage in computation performance; when integrated into a farm management system, it needs a certain cost of computation resources, such as high-performance GPUs and databases, but this is worthwhile because the knowledge of dairy cow disease is relatively stable, and the model does not require being rerun for many times. Furthermore, when implementing our model or the NER system in real-world settings, the size and quality of datasets are important factors, especially in real-world medical reports written by specialists, and spending a large amount of time on data cleaning and annotation is required, which limits its application in real-world scenarios.

## 5. Conclusions

In this paper, we proposed a Chinese named entity recognition model for dairy cow diseases by the fusion of multi-semantic features and a self-attention mechanism. We extracted character-level, pinyin-level, glyph-level, and lexical-level features to tackle the problems of homophony characters with different structures, hieroglyphic characters with a certain meaning, and complex characters with a mix of special terms that commonly exist in the dairy cow disease corpus and used a Bi-LSTM network based on multi-head self-attention to capture long-distance dependency while focusing on characters that are important for entity recognition. Experimental results show that the F1 score of our proposed model reached 92.18% with a precision and recall of 91.93% and 92.42%, respectively. Our model outperformed the baselines and models from related works and was demonstrated to be suitable for the dairy cow disease corpus. However, our model still has problems that need to be improved, as follows: (1) It was demonstrated in our experiments that pre-trained large language models have advantages in character-based works. Therefore, we will employ pre-trained models like BERT and RoBERTa to extract the character-level features, thereby improving the performance of our model. (2) In addition to the common features of the entities mentioned in this paper, there are also a large number of nested entities in the dairy cow disease corpus, e.g., “牛皮蝇蛆病 (Cattle Hypodermosis)”, which is an entity of a disease name but contains an entity of the pathogen “牛皮蝇 (Hypoderma bovis)”. We will explore solutions to address this challenge in our future work. (3) Our model still has room for improvement in computation performance, and we aim to explore more lightweight methods to reduce the computational burden of our model.

## Figures and Tables

**Table 1:** Parameters of our proposed PGLA-DCNER model.

Parameters	Value
Dimension of character vectors	50
Dimension of word vectors	50
Dimension of hidden layer in Bi-LSTM	300
Number of layers in Bi-LSTM	1
Dimension of hidden layer in attention	600
Number of heads in attention	10
Dropout rate	0.5
Learning rate	0.002
Batch size	16
Max. epoch	80
Optimization algorithm	Adam

**Table 2:** An overview of the models compared in the experiments.

	Model	Encoder	External Features			Attention Mechanism
			Lexicon	Pinyin	Glyph	
baselines	SoftLexicon(CNN)	CNN	√	×	×	×
	SoftLexicon(Transformer)	Transformer	√	×	×	×
	SoftLexicon(Bi-LSTM)	Bi-LSTM	√	×	×	×
ablation models	+pinyin	Bi-LSTM	√	√	×	×
	+glyph	Bi-LSTM	√	×	√	×
	+attention	Bi-LSTM	√	×	×	√
	+pinyin+glyph	Bi-LSTM	√	√	√	×
	+pinyin+attention	Bi-LSTM	√	√	×	√
	+glyph+attention	Bi-LSTM	√	×	√	√
our model	PGLA-DCNER	Bi-LSTM	√	√	√	√
related works	Bi-LSTM-CRF	Bi-LSTM	×	×	×	×
	Bi-LSTM-Attention-CRF	Bi-LSTM	×	×	×	√
	BERT-Bi-LSTM-CRF	Bi-LSTM	×	×	×	×
	RoBERTa-Bi-LSTM-CRF	Bi-LSTM	×	×	×	×
	WC-LSTM	Bi-LSTM	√	×	×	×
	LR-CNN	CNN	√	×	×	×
	FLAT	Transformer	√	×	×	×
	MECT	Transformer	√	×	√	×

Notes: ‘√’ represents using components such as “Lexicon” or “Pinyin” in a model. ‘×’ represents that do not use components such as “Lexicon” or “Pinyin” in a model.

**Table 3:** Evaluating results of baseline models, related works’ models, and our proposed model.

	Models	Precision (%)	Recall (%)	F1 (%)
baselines	SoftLexicon (CNN)	75.53	86.18	80.50
	SoftLexicon (Transformer)	83.41	88.65	85.95
	SoftLexicon (Bi-LSTM)	85.76	93.88	89.64
character-based models	Bi-LSTM-CRF	84.70	90.94	87.71
	Bi-LSTM-Attention-CRF	84.69	92.07	88.23
	BERT-Bi-LSTM-CRF	87.48	92.29	89.82
	RoBERTa-Bi-LSTM-CRF	86.33	92.65	89.38
ensemble models	WC-LSTM	88.03	89.54	88.78
	LR-CNN	84.54	93.44	88.77
	FLAT	86.86	94.56	90.54
	MECT	86.21	95.35	90.55
our model	PGLA-DCNER	91.93	92.42	92.18

**Table 4:** Ablated experiment results of our model.

	Models	Precision (%)	Recall (%)	F1 (%)
baseline	SoftLexicon	85.76	93.88	89.64
ablation models	+pinyin	88.58	92.96	90.72
	+glyph	86.49	93.83	90.01
	+attention	89.66	91.28	90.47
	+pinyin+glyph	88.81	93.44	91.06
	+pinyin+attention	89.55	92.55	91.02
	+glyph+attention	88.56	93.03	90.74
our model	+pinyin+glyph+attention	91.93	92.42	92.18

**Table 5:** Results of PGLA-DCNER with different glyph features.

	Models	Precision (%)	Recall (%)	F1 (%)
1	Radical_CNN	91.12	92.34	91.73
2	Tianzige_CNN	90.16	92.58	91.36
3	GLYNN	91.93	92.42	92.18

**Table 6:** Training time, inference speed, and memory usage of different models.

Models	Training Time (s/iter)	Inference Speed (s/iter)	Memory Usage (G)
SoftLexicon	129.64	17.75	14.58
Bi-LSTM-CRF	126.09	17.41	14.28
Bi-LSTM-Attention-CRF	129.00	17.68	14.27
BERT-Bi-LSTM-CRF	367	31	6.43
RoBERTa-Bi-LSTM-CRF	349	36	6.44
WC-LSTM	186.62	32.83	9.92
LR-CNN	196.71	23.24	6.42
FLAT	120.3	12.85	10.89
MECT	106	10.25	4.63
PGLA-DCNER	130.41	17.85	16.02

## Data Availability

The data presented in this study are available on request from the corresponding author.
